# Applying the FMR Technique to Analyzing the Influence of Nitriding on the Magnetic Properties of Steel

**DOI:** 10.3390/ma15124080

**Published:** 2022-06-08

**Authors:** Slawomir Maksymilian Kaczmarek, Jerzy Michalski, Grzegorz Leniec, Hubert Fuks, Tadeusz Frączek, Agata Dudek

**Affiliations:** 1 Department of Technical Physics, Faculty of Mechanical Engineering and Mechatronics, West Pomeranian University of Technology in Szczecin, al. Piastów 48, 70 311 Szczecin, Poland; grzegorz.leniec@zut.edu.pl (G.L.); hubert.fuks@zut.edu.pl (H.F.); 2 Faculty of Production Engineering and Materials Technology, Częstochowa University of Techology, 42 201 Częstochowa, Poland; michalski@imp.edu.pl (J.M.); tadeusz.fraczek@pcz.pl (T.F.); dudek@wip.pcz.pl (A.D.)

**Keywords:** AISI steel, nitriding, FMR, SQUID

## Abstract

This paper presents the relationship between the chemical composition and size of steel balls, the parameters of the nitriding process, and their magnetic properties, defined in this study by ferromagnetic resonance (FMR) and SQUID. Balls made from AISI 1010 and AISI 52100 steels, with diameters of 2.5 and 3 mm, respectively, were investigated. On samples made of AISI 1010 and AISI 52100 steel, single-phase layers of iron nitrides γ’ with a thickness of g_mp_ = 50 and 37 μm, respectively, were produced. Then, the samples were annealed at a temperature of 520 °C for 4 h in an inert atmosphere (N_2_/Ar) at a pressure of 200 Pa. After the nitriding processes, steel balls were subjected to standard physical metallurgy and X-ray examinations. During annealing of nitrided layers with a two-phase layer of iron nitrides, at first, the transformation of the ε phase into the γ’ phase with the release of nitrogen into the atmosphere takes place. The FMR signals did not originate from isolated ions, but from more magnetically complex systems, e.g., Fe–Fe pairs or iron clusters, while the observed FMR line position is normally even lower and occurs for a magnetic induction below 200 mT. The fact that the magnetic centers did not contain mainly isolated Fe ions, additionally confirmed the abnormal increase in resonance signal intensity as a function of temperature, which is a behavior inconsistent with the Curie–Weiss law. The results obtained from measurements by the SQUID method, recording variations in magnetization as a function of temperature, confirm the untypical reinforcement of the magnetic conditions of the samples with the increase in temperature. For the samples tested, the magnetization was relatively weaker when the tests were conducted in a stronger magnetic field.

## 1. Introduction

In the 1920s, Lehrer was the first to conduct research on the stability of iron nitrides depending on temperature and the nitrogen potential [1]. The research on the durability of iron nitrides initiated by Lehrer is systematically continued both in nitriding and inert atmospheres. Phase transformations of iron nitrides are a natural phenomenon in the nitriding process and may occur in the heating and cooling stage and at a constant temperature as a result of changes in the value of the nitrogen potential [2]. In the initial stage of the nitriding process, a solid solution of nitrogen in alpha iron is formed. After exceeding the limit of nitrogen solubility in iron, nuclei of the γ’-Fe_4_N_1−x_ phase are formed on its surface, followed by their coalescence and the growth of the monophasic layer γ’ [3]. If the nitrogen potential reaches levels corresponding to the stability of the ε-Fe_3-z_N phase, ε phase nuclei are formed on the surface of the γ’ layer, and their coalescence is followed by the growth of the γ’/ε layer. In the next stage of the process, porosity in the near-surface zone of the iron nitride layer is formed, related to the instability of iron nitrides at a pressure of 1 atm. according to the Fe-N_2_ equilibrium system [2].

The stability range of iron nitrides, like the Lehrer system, also describes the Fe-N phase equilibrium system [4]. It shows that γ’-Fe_4_N_1−x_ nitride is almost stoichiometric at ambient temperature, while ε-Fe_2_N_1−x_ nitride has a wide range of homogeneity. The Fe-N equilibrium diagram of pure components at a pressure of 0.1 MPa results in a very low solubility of nitrogen in ferrite; the formation of iron nitrides requires a nitrogen pressure of a few GPa. Such conditions can be achieved at a physical pressure of 0.1 MPa in a mixture of ammonia-hydrogen gas (NH_3_/H_2_). The metastability of iron nitrides in relation to the Fe-N system is similar to the metastability of cementite in relation to the Fe-C system [2].

Under normal conditions, iron nitrides are unstable and should decompose to give gaseous nitrogen [5]. The loss of nitrogen into the atmosphere is irreversible. According to [6], the equilibrium pressure of gaseous nitrogen for Fe_4_N nitride is approximately 1500 atmospheres. However, it should be emphasized that the rate of nitrogen removal from the crystal lattice depends on the diffusion of nitrogen atoms to the surface [6]. The high activation energy of the N + N→N_2_ reaction makes it difficult to denitrify; therefore, iron nitrides are stable up to approximately 420 °C.

The authors of [7] investigated the phase transformations of the mixture of ε and γ’ phases at a temperature range of 340–420 °C. They found that up to a temperature of 360 °C, the share of the γ’ phase and nitrogen concentration in the ε phase on the border with the γ’ zone increased. Continuing annealing at temperatures of up to 420 °C, the authors found nitrogen diffusion from zone ε through zone γ’ followed by a reaction with the ferrite of the substrate, as a result of which a new phase of γ’ is formed. As such, the total thickness of the iron nitride layer increases, but no nitrogen is emitted to the atmosphere.

The authors of [5] found that heating of the nitrided iron powder in vacuum to 400 °C does not cause any phase transformation. For all the phases detected (α-Fe, γ’-Fe_4_N, and ε-Fe_2__–3_N), only thermal expansion of the lattices is observed in this temperature range. Starting with 400 °C, they observed lowering of the parameters of the cells of the ε-Fe_2__–3_N phase due to the decrease in the content of nitrogen in this phase. At the same time, a decrease in the intensity of reflections of the ε phase from 500 °C was observed, until their full disappearance at 560 °C and simultaneous growth in the intensity of the lines of γ’reflect absorption of the ε-nitride by the γ’-phase in the heating process. In [8], a thermogravimetric study was carried out under continuous heating at a rate of 30 K/min in an argon atmosphere at 20–1100 °C, with the results showing that the most thermally stable materials from the standpoint of preservation of nitrogen are powders with a developed ε phase surface layer. A very high temperature range for the start of denitrification (820–850 °C), at a mean rate of nitrogen emission equal to 0.8–0.9 wt.%/min, was detected for powders nitrided for 5 and 8 h, containing 37 and 18 wt.% ε-nitride in the surface layers, respectively. The results for the powder nitrided for 48 h proved that the γ’-nitride is the most thermally unstable compound, at a mean rate of nitrogen emission of 4.8 wt.%/min.

In papers on nitriding treatments applied to iron and its alloys, changes in their mechanical properties (hardness and brittleness) and chemical properties (corrosion resistance and oxidation), which they obtain after the nitriding process, are most often discussed. The listed properties of the iron nitride layer depend on its thickness and phase composition. In gas nitriding processes, the phase composition of the iron nitride layer is regulated by the nitrogen potential, and the thickness by the process time.

During the nitriding process, the iron nitride layer and the diffusion zone grow simultaneously. Ratajski [9] proved that the kinetics of the increase in the thickness of the diffusion zone of the nitrided layer is significantly influenced by the phase composition of the iron nitride layer. If a layer of iron nitride γ’ is formed on the steel surface, the kinetics of the increase in the diffusion zone thickness will be lower than the kinetics of the increase in the thickness of the diffusion zone when there is a layer of iron ε nitrides on the steel surface. The authors of [10] also noted the significant influence of the phase composition of the iron nitride layer on the kinetics of the increase in the diffusion zone thickness of the nitrided layer. The results of the above-mentioned studies do not clearly indicate that the iron nitride layer is a nitrogen source for the growing diffusion zone of the nitrided layer.

The discussed research was aimed at clarifying whether, during annealing of nitrided steels in an ammonia-free atmosphere, nitrogen emission from the nitrided layer is accompanied by a change in the thickness of the surface layer of iron nitrides and the diffusion zone. We also aimed to determine changes in the magnetic properties of the investigated steel balls that could be assigned to the emission of nitrogen from the nitrided layer. The aim of these investigations is also to determine correlations between the magnetic properties and the structure of the balls that underwent the nitriding process, determined using the FMR and SQUID techniques. Such an approach is known in the literature [11]. After describing the methodology in Section 2, where investigated materials and their physical and chemical properties are described, we present mechanical, FMR and magnetic susceptibility SQUID results and their discussion in Section 3. In Section 4, conclusions are presented.

## 2. Research Methodology

The non-alloy steel AISI 1010 and the alloy steel AISI 52100 were subjected to the nitriding and annealing processes. AISI 1010 steel, in addition to carbon, contains only ordinary admixtures, while AISI 52100 steel, in addition to carbon and ordinary admixtures, contains 1.5 wt.% chromium. Chromium is an element with a high affinity for nitrogen. The use of these steels made it possible to investigate how the presence of chromium in the steel will affect the kinetics of the increase in the thickness of the layer of iron nitrides during nitriding and their decomposition during annealing. The chemical composition of the steel and the diameters of the samples in the form of a sphere are given in Table 1. The samples were constantly subjected to gas nitriding processes, and then some of the nitrided samples were annealed at 520 °C within 4 h in an inert atmosphere (N_2_/Ar) at a pressure of 200 Pa. The parameters of the nitriding and annealing processes are given in Table 2.

Samples were annealed before and after the process. Mass was measured to an accuracy of 10^−5^ g. Depending on the diameter of the ball, 15 to 100 balls were annealed in one process. Samples of different diameters were used, and the loss in mass of samples was related to a unit area.

Measurements of hardness distributions of nitrided, nitrided and annealed layers were made using the Vickers method on a semi-automatic FM 7 hardness tester from FUTURE-TECH. at a load of 100 G (980.7 mN).

The phase composition of the iron nitride layer on the samples was determined by X-ray diffraction using the Seifert 3003TT X-ray diffractometer, using K_α_Co radiation and symmetrical measurement geometry (see Table 3). The measurement parameters were: voltage 30 kV, current 40 mA, step 2θ 0.05°, and counting time 5 s. The range of recorded diffraction angles 40÷58° included the main characteristic lines from iron nitrides γ’ and ε and from the steel substrate, according to the patterns from the PDF4+ diffractometric database.

The iron nitride layer thickness assessment method developed and used in this paper has been described elsewhere [11].

FMR spectra were recorded at a temperature range of 82–300 K using a conventional X-band spectrometer ELEXSYS E500 operating at 9.46 GHz and 0.62 mW microwave power. The first derivative of the absorption spectrum was recorded as a function of the applied magnetic induction, which ranged from 0 to 1.4 T. The EPR/NMR program was used to determine spin-Hamiltonian (SH) parameters and a local symmetry of the paramagnetic ions [12]. Optimization and normalization of the parameters were made using the root-mean-squared deviation method. Due to the FMR signal asymmetry of the investigated samples, Dysonian line shape was adopted. The shapes of the FMR spectra could be described by a function proposed by Dyson, where the line shape is broadened due to a dispersion phenomenon [11,12,13]. It is valid especially in conducting materials, such as metals, semimetals, semiconductors. Temperature dependences of FMR spectra were analyzed taking into account three parameters: FMR magnetic susceptibility, χ_FMR_, square of magnetic moment χ_FMR_ *T, and the position of resonance line, *g*. FMR magnetic susceptibility is equal to double integral of the measured FMR spectra. It corresponds, generally, to magnetic susceptibility, measured by the SQUID technique, but there appear to be some differences due to differences in the procedures applied. Magnetic susceptibility measurements obey all of magnetic ions present in the investigated sample, while the FMR technique only detects active ions, e.g., iron. Static magnetic susceptibility measurements were performed using a Superconducting QUantum Interference Device (SQUID) MPMS-XL7 magnetometer. The measurements were recorded at a temperature range from ~50 K to room temperature at a magnetic field of 100 Oe. Hysteresis loops were also measured to determine other magnetic properties, such as H_c_, coercive field and B_r_, remnant parameters.

## 3. Results and Discussion

### 3.1. Mechanical Properties

**Figure 1:**
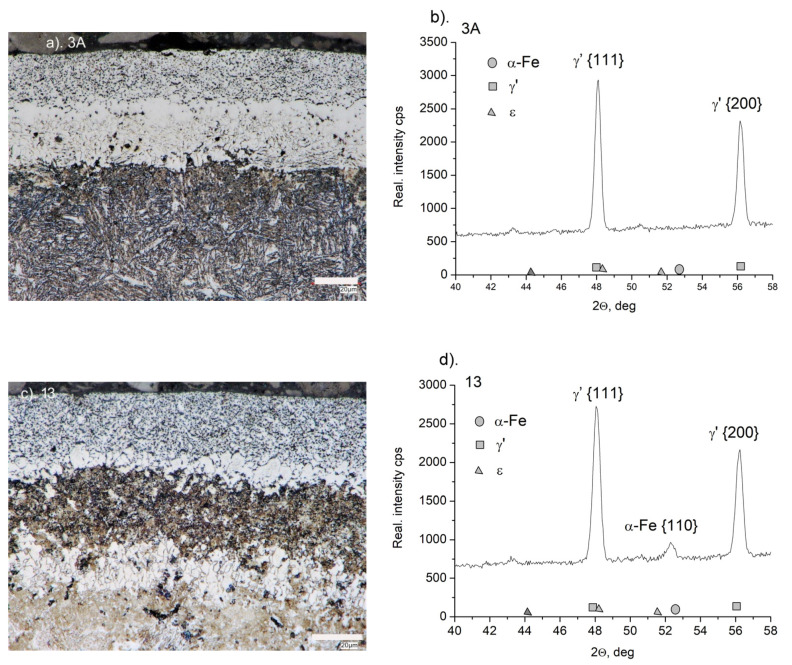
(**a**) Microstructure of the nitrided layer on AISI 1010 steel (sample 3A) and nitrided and annealed steel (sample 13) (**c**). Diffractograms from the surface of nitrided samples (sample 3A) (**b**) and nitrided and annealed samples (sample 13) (**d**).

On the AISI 1010 steel (sample 3A), a monophasic layer of iron nitrides γ’ with a thickness of g_mp_ = 50 ± 1 μm was formed during the nitriding process. The layer of iron nitrides is of uniform thickness, and a clear boundary between the layer and the substrate is visible (Figure 1a).

On the diffractogram of the 3A sample, only the lines characteristic of γ’-Fe_4_N iron nitrides, which occur at a range of 2θ angles under study, were recorded (Figure 1b). In the iron nitride layer, two zones can be distinguished, lighter than the substrate, with a thickness of approximately 30 μm, and a darker porous zone (g_por_), with a thickness of 20 μm near the surface zone of the layer (Figure 1a).

**Figure 2:**
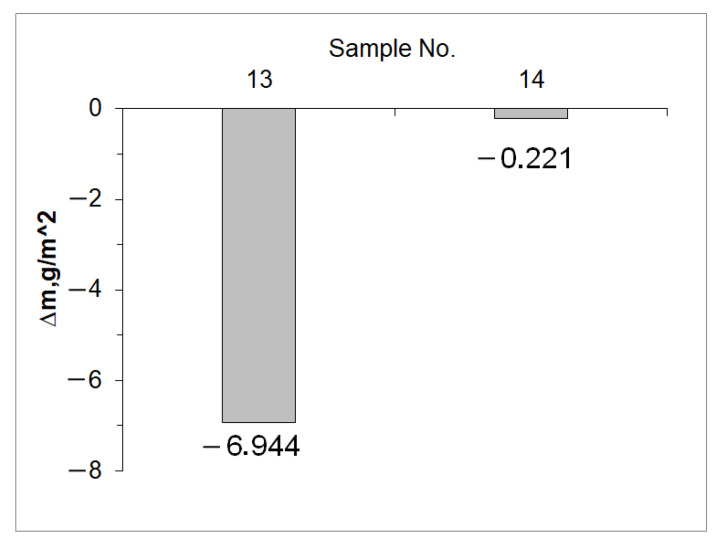
Change in sample weight next after annealing (g/m^2^).

The structure of the near-surface zone of the iron nitride layer after annealing (sample 13) is clearly different from the structure of sample 3A. The thickness of the porous zone increased from 20 to 26 μm, and also increased in porosity (Figure 1c). A significant part of the non-porous zone (the black area directly below the porous zone) was decomposed. The intensity of the lines, characteristic of the planes γ’ {111} and γ’ {200} decreased. On the diffractogram, there is a line characteristic of the α-Fe substrate, and its appearance may be associated with the disappearance of part of the non-porous zone of iron nitrides (Figure 1d). The disappearance of this zone also explains the large loss in mass of the samples after annealing, compared to other samples (see Figure 2).

**Figure 3:**
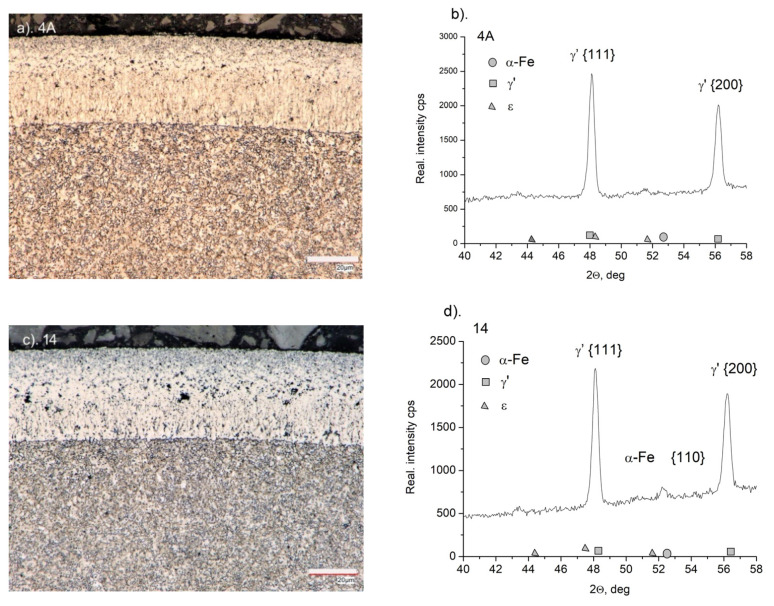
(**a**) Microstructure of the nitrided layer on AISI 52100 steel (sample 4A) and nitrided and annealed steel (sample 14) (**c**). Diffractograms from the surface of nitrided samples (sample 4A) (**b**) and nitrided and annealed samples (sample 14) (**d**).

**Figure 4:**
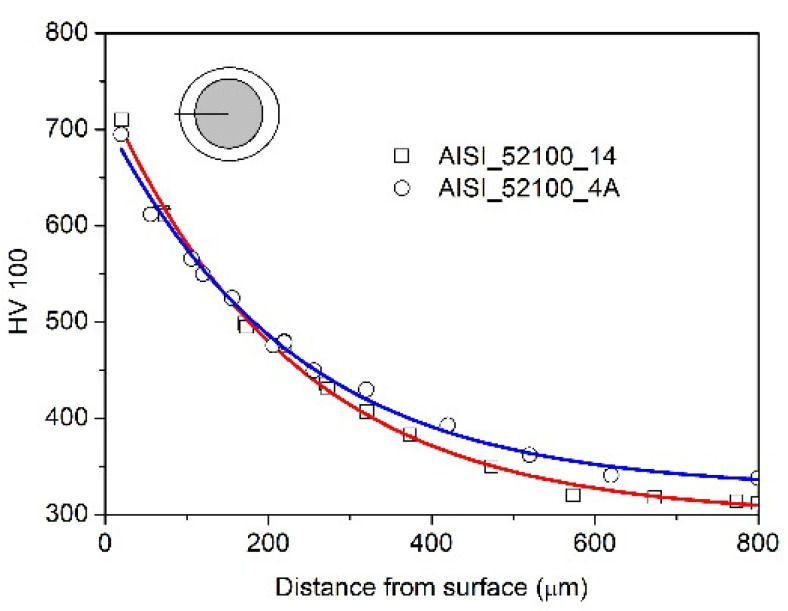
Distribution of the hardness (cross-section through the ball) in the nitrided layer (4A) and nitrided and annealed steel (14).

On AISI 52100 steel (sample 4A), a monophasic layer of iron nitrides γ’ with a thickness of g_mp_ = 37 ± 1 μm was formed during the nitriding process (Figure 3a). The layer of iron nitrides is of uniform thickness, and a clear boundary between the layer and the substrate is visible. On the diffractogram of sample 4A, only the lines characteristic of γ’-Fe_4_N iron nitrides, which occur at a range of 2θ angles under study, were recorded (Figure 3b). The formed layer of iron nitrides is thinner than that obtained on the 3A sample in the same process. With AISI 1010 non-alloy steel, from which the 3A sample was made, due to the low solubility of nitrogen in iron, the vast majority of nitrogen from the nitriding atmosphere builds a layer of iron nitrides. Alloy steel AISI 52100 contains chromium, which has a high affinity for nitrogen. Nitrogen from the nitriding atmosphere participates in the construction of the iron nitride layer as well as in the construction of the solution layer, which develops directly under the iron nitride layer, increasing the hardness of the steel in the diffusion zone of the nitriding layer (Figure 4).

After the annealing process, the structure of the steel (sample 14) did not change significantly (Figure 3c). The microstructure shows a layer of iron nitrides with a thickness of g_mp_ = 38 ± 1 μm, with a less pronounced columnar zone closer to the substrate. As a result of the denitrification of the iron nitride layer in the porous zone, the pores are larger than in the nitrided layer (4A) (Figure 3a). The intensity of the characteristic lines from the planes γ’ {111} and γ’ {200} decreased and the characteristic line α-Fe {110} indicates that in the case of sample 14 (Figure 3c), the radiation analyzes the substrate as opposed to the nitrided sample only (sample 4A) (Figure 3a). A slight loss in sample weight after annealing indicates that the γ’ phase life on alloy steel is greater than that on non-alloy steel (Figure 2).

The hardness distribution on the cross-section of the sample of AISI 52100 nitrided and nitrided and annealed steel is almost the same; this proves that the iron nitride layer cannot be a source of iron for the diffusion layer (Figure 4).

### 3.2. FMR Spectra

**Figure 5:**
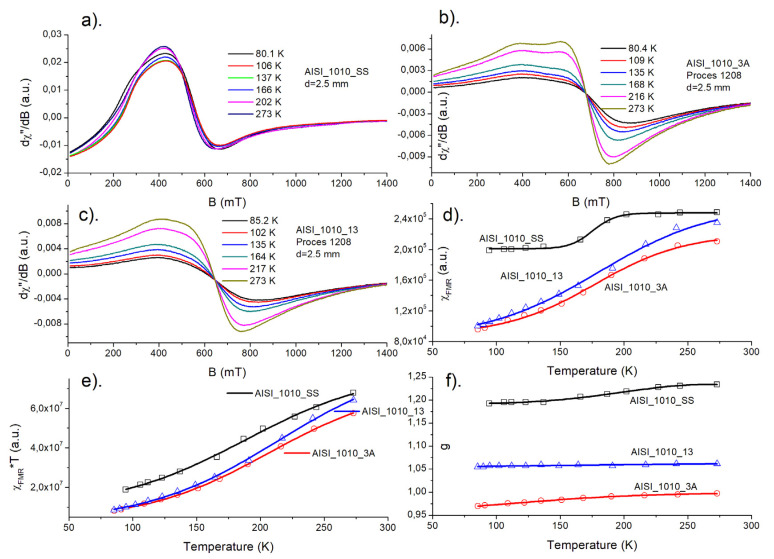
AISI_1010 sample. (**a**–**c**) Temperature dependence of FMR spectra for starting sample (SS), nitrided (3A), and nitrided and annealed steel (13), respectively; (**d**–**f**) temperature dependences of FMR magnetic susceptibility, χ_FMR_, square of magnetic moment, χ_FMR_ *T, and resonance position of FMR signal, *g*, respectively.

**Figure 6:**
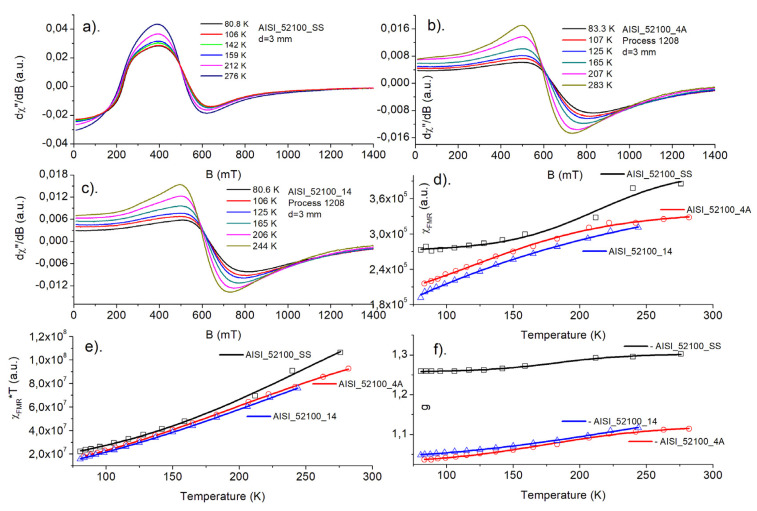
AISI_52100 sample. (**a**–**c**) Temperature dependence of FMR spectra for starting sample (SS), nitrided (4A), and nitrided and annealed steel (14), respectively; (**d**–**f**) temperature dependences of FMR magnetic susceptibility, χ_FMR_, square of magnetic moment, χ_FMR_ *T, and resonance position of FMR signal, *g*, respectively.

The magnetic resonance signal of steel balls (Figure 5 and Figure 6) contains a very wide, intense line observed at an accessible temperature range of 80–300 K. Due to the high density of responsible magnetic centers, such an experiment should be treated as FMR rather than electron paramagnetic resonance (EPR) experiment. Analyzing the overall shape of the resonance lines, one can see, that the “non-modified” (SS—starting samples) ball materials usually possess a very complex FMR signal, being far from a standard Lorentzian function. The asymmetry of the resonance signal could be explained by the very high conductivity of steel, leading to creation of skin currents in an FMR experiment. Such a phenomenon was described by Dyson [13] and examined further in an EPR experiment [14,15]. According to the theory, the symmetric Lorentzian line is distorted due to the non-uniform distribution of the microwave field in the sample. The level of asymmetry, among many other factors, depends on the relation between the size of a sample and skin depth. The contribution of the asymmetric Dyson-like function to the overall FMR line is expected to be weaker for smaller samples. Thermal treatment (nitriding, annealing) leads to the increasing homogeneity of magnetic entities, which can be seen from the increasing symmetry of the observed FMR signal compared to the signal of non-modified samples.

In the Figure 5a–c and Figure 6a–c, temperature dependence of FMR spectra is shown for starting sample (SS), nitrided (3A for AISI_1010 and 4A for AISI_52100 steels), and nitrided and annealed steel (13 for AISI_1010 and 14 for AISI_52100 steels), respectively. As one can see, a shape of FMR signal changes significantly, when successive process is applied.

In the Figure 5d–f and Figure 6d–f, temperature dependences of FMR magnetic susceptibility, χ_FMR_, square of magnetic moment, χ_FMR_ *T, and resonance position of FMR signal are presented, respectively. Magnetic susceptibility (d) increases for all samples with temperature, although higher values are observed for SS samples. Magnetic moments (e) decrease with decreasing temperature, which suggests antiferromagnetic-like interactions dominating magnetic behavior of the samples. The resonance position of FMR lines, *g*, (f) shifts towards lower values both for nitrided and annealed samples.

**Figure 7:**
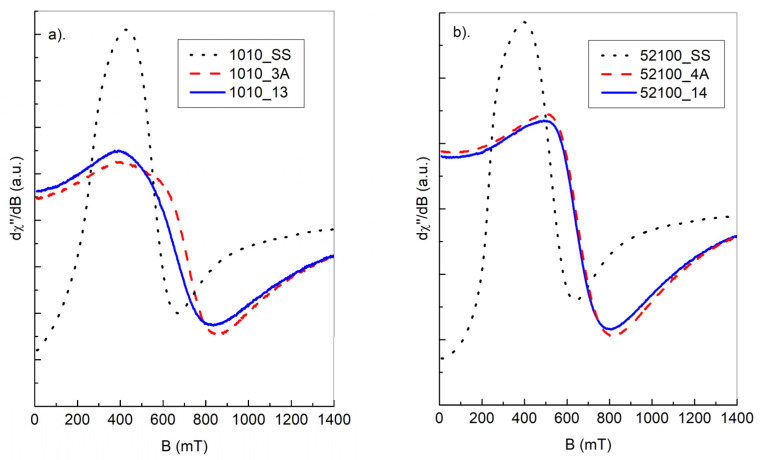
FMR signals of the following samples: starting sample (SS), nitrided (3A or 4A), nitrided and next additionally annealed (13 or 14), measured for AISI 1010, *Φ* = 2.5 mm (**a**) and AISI 52100, *Φ* = 3 mm (**b**).

Although the above temperature dependences are obtained only in the 80–300 K ranges, our previous investigations in the 3–300 K range, concerning the same samples tested for something different thermodynamical conditions, confirmed the above conclusions [16]. Comparison of the FMR spectra of balls subjected to different heat treatments is shown in Figure 7a,b. As shown, thermal processes lead to a more symmetric FMR resonance line, indicating the increasing homogeneity of responsible magnetic centers.

As was mentioned earlier, the overall FMR signal could be treated as a superposition of two resonance lines: Lorentzian component, representing the uniform distribution of iron centers in dense magnetic system, and the Dysonian component, representing the share of conducting electrons in, e.g., the skin current effect. The following figures show the decomposition of the overall resonance signal into two components: Lorentzian and Dysonian like. As the Dysonian line represents the contribution of free electrons, its position should be fixed close to the spectroscopic factor *g* = 2.

**Figure 8:**
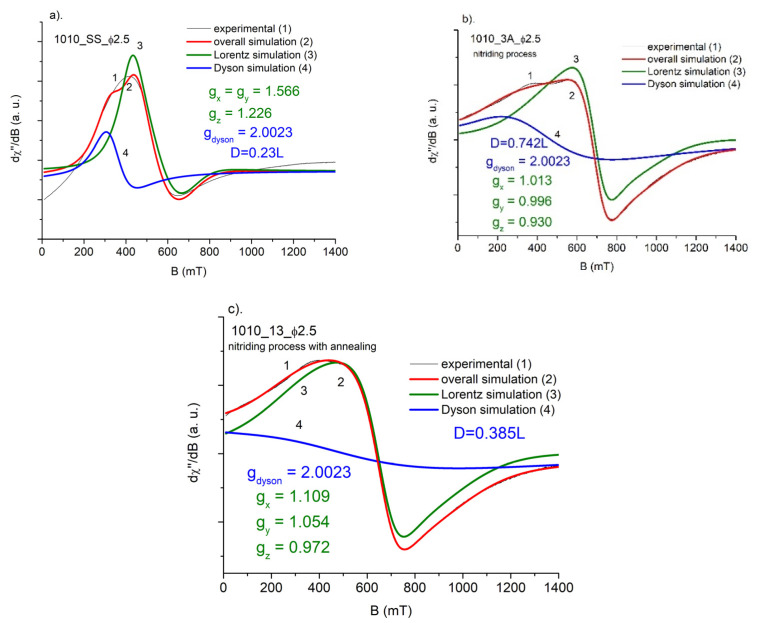
Simulation of the FMR spectra of AISI 1010: SS, 3A, and 13 balls: (**a**). Starting sample (SS), (**b**) nitrided (3A), and (**c**) nitrided and annealed (13). Ball diameter *Φ* = 2.5 mm. *g_i—_*simulated parameters of *g*-matrix. *D—*the ratio of the signal coming from the Dyson line to the signal coming from the Lorentz line.

**Figure 9:**
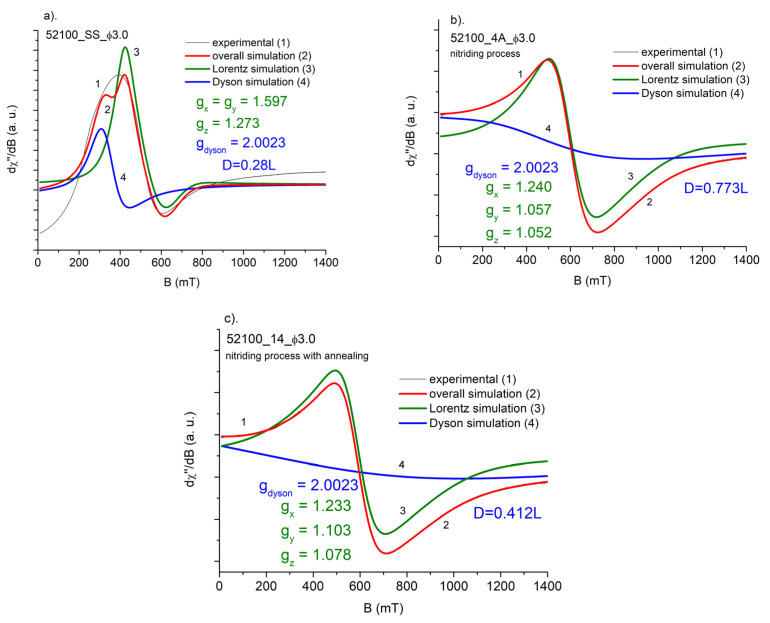
Simulation of the FMR spectra of AISI 52100: SS, 4A, and 14 balls: (**a**). Starting sample (SS), (**b**) nitrided (4A) and (**c**) nitrided and annealed (14). Ball diameter *Φ* = 3 mm. *g*_i—_simulated parameters of g-matrix. *D*—the ratio of the signal coming from the Dyson line to the signal coming from the Lorentz line.

As shown, the results of simulation are far from satisfactory for starting samples (Figure 8a and Figure 9a). This reflects the complex nature of iron signals in this case. Responsible magnetic centers are far from uniformly distributed, especially in the starting samples. So, the Lorentzian model does not work properly. Contrary to this, the FMR signal of the balls next to nitriding and further annealing could be properly described by single Dysonian and Lorentzian lines (Figure 8b,c and Figure 9b,c). Thus, both thermal processes improve the level of homogeneity of magnetic iron centers in starting samples. As we mentioned earlier, the existence of Dysonian line reflects the existence of asymmetric component in Lorentzian distribution arising from nonuniform absorption of microwave in resonance experiment, usually connected with generation of skin currents in a sample. This non-uniform distribution may be represented by a so-called *alpha* parameter, included in the Dysonian function. In our case, a higher share of skin currents means that *alpha* tends to a value of 1, whereas *alpha* = 0 for simple absorption process, when the Dysonian function goes to Lorentzian shape [16]. As one knows, for smaller-diameter balls the skin effect is expected to be weaker, thus the *alpha* parameter should be less than 1. In the case of two types of balls, we could not see this effect, partly due to a small difference between diameters and the very high width of the measured Dysonian line.

We also performed studies of the integrated intensity of the FMR signal, taken as the area under the FMR absorption curve, in order to split FMR signal into a Dyson line and a Lorentz line. The ratio of the signal coming from the Dyson line (*D*) to the signal coming from the Lorentz line (*L*) turned out to be equal *D* = 0.23*L* for 1010_SS sample. It corresponds to a 23% contribution of free electrons (skin effect) to the total FMR signal intensity. Almost the same is true for 52100_SS sample. The Dyson line contribution is *D* = 28*L*. However, the ratio of overall integrated intensities the 2.5 mm diameter 1010_SS and the 3.0 mm diameter 52100_SS balls is 18.6%. It is worth noting that the ratio of the diameters of both balls is equal to 0.83(3), i.e., the difference between them is 16.6(6)% (see Table 4). This is a certain indication to check (for other diameters) the possibility of determining the thickness of the nitrided layer on the basis of the analysis of the integral intensity of the FMR spectrum. This is partly confirmed by a previous FMR study [16]. In the nitriding process, the contribution of Dyson lines to the total intensity of the FMR signal significantly increases–for 1010_SS steel to a value of 74.2%, while for 52100_SS steel to a value of 77.3%. The annealing process reduces these values to 38.5% and 41.2%, respectively.

It is also worth noting that the symmetry of the closest surroundings of the iron ions changes (see Figure 8 and Figure 9) next to subsequent thermal process. When one applies the following Spin Hamiltonian:

H = *µ*_B_*B*∙*g*∙*S*

where *µ_B_*—Bohr magneton, *B*—induction of magnetic field, *g*—spectroscopic splitting matrix, and *S*—electron spin of Fe, to starting sample, we can detect higher (g_x_ = g_y_ ≠ g_z_) symmetry of Fe ions than for a steel next to nitriding and/or annealing (g_x_ ≠ g_y_ ≠ g_z_). This change in a system symmetry may be due to the appearance of an additional Fe_4_N phase introduced by nitriding.

### 3.3. Magnetic Susceptibility Measurements

**Figure 10:**
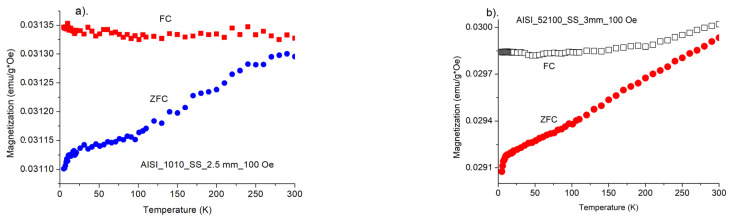
(**a**). Magnetic susceptibility of AISI_1010_SS sample in a range 3–300 K for H = 100 Oe, (**b**). Magnetic susceptibility of AISI_52100_SS sample in a range 3–300 K for H = 100 Oe.

Figure 10a,b shows magnetization measurements of starting samples for AISI_1010 and AISI_52100 steels. As can be seen the samples characterize large magnetic anisotropy and unusual temperature dependence in a 3–300 K temperature range. The measurements were performed at FC and ZFC modes. For the investigated samples, we observed that the magnetization was slightly weaker, when the investigations were performed in a stronger magnetic field.

So, the results obtained from the SQUID method for magnetization versus temperature confirm the untypical behavior of the magnetization with the increase in temperature. This was also observed previously in FMR measurements.

**Figure 11:**
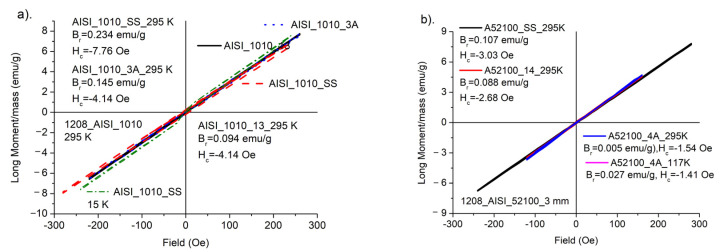
(**a**). Hysteresis loops for AISI_1010 samples, (**b**). Hysteresis loops for AISI_52100 samples. H_c_ and B_r_—coercive field and remnant parameter, respectively.

Figure 11 indicate that all investigated samples reveal ferromagnetic interactions, independently on a kind of process for temperatures 15–295 K. Only small differences are observed for different processes. The increase in magnetization, as well as in FMR magnetic susceptibility, with temperature, could be observed in an antiferromagnetic structure. According to former reports concerning similar kind of steel [17] we are dealing with magnetic material, in which a minority of ferromagnetic components occurs as an uncertainty in general antiferromagnetic structure. Increasing in temperature destroys antiferromagnetic ordering and magnetization is enhanced. Similar behavior of magnetization with temperature relation was observed in other Fe-containing materials [18], where increase in the magnetization was explained by the creation of Fe^2+^ ions in the structure built of Fe^3+^.

## 4. Conclusions

Based on the obtained results, it can be concluded that during annealing of nitrided layers with a two-phase layer of iron nitrides, at first, the transformation of the ε phase into the g’ phase with the release of nitrogen into the atmosphere takes place. This process is accompanied by a loss of sample mass, but this does not change the thickness of the iron nitride layer to the extent that can be determined by standard metallographic tests. The FMR technique is sensitive enough to detect differences in the physical properties of AISI steel underwent to different kinds of the nitriding and annealing processes. 

The FMR spectrum of the investigated steel balls can be divided into two parts—Lorentzian like and Dysonian like. The former is responsible for iron ions, while the latter for free electrons. The overall FMR spectrum is a superposition of the two lines. Samples with a higher carbon content possess more symmetric FMR signals and higher intensity of the Dyson-like line. It is assigned to a share of free electrons in FMR line-shape. The ratio of the signal coming from the Dyson line to the signal coming from the Lorentz line turned out to be equal *D* = 0.23*L* for 1010_SS sample and *D* = 0.28*L* for 52100_SS sample. In the nitriding process, the contribution of Dyson lines to the total intensity of the FMR signal significantly increases—for 1010_SS steel to a value of 74.2%, while for 52100_SS steel to a value of 77.3%. The annealing process reduces these values to 38.5% and 41.2%, respectively.

Temperature dependences of FMR spectra show untypical behavior. FMR magnetic susceptibility increases for all samples with temperature, although the higher values are observed for SS samples. Magnetic moments decrease with decreasing temperature, which suggests antiferromagnetic-like interactions dominating magnetic behavior of the samples.

The above conclusions were repeated in magnetization measurements. So, in a case of our steels, a minority of ferromagnetic components, as, e.g., carbon, occurs as an imperfection in general antiferromagnetic structure.

## Figures and Tables

**Table 1:** Chemical composition of steels used in the tests.

Grade Steel	Sample No.	Φ (mm)	Element Content in wt.%						
			C	Mn	Si	P	S	Ni	Cr
AISI 1010	3A; 13	2.50	0.10	0.5	01	0.04	0.05	-	-
AISI 52100	4A; 14	3.00	1.0	0.4	0.3	0.02	0.02	-	1.5

**Table 2:** The basic parameters of the nitriding annealing processes.

	Parameters of the Nitriding Processes (1208)							Parameters of the Annealing Processes		
Sample No.	Stage I			Stage II			Inlet Atmosphere	T [°C]	t [h]	Inlet Atmosphere
	T [°C]	t [h]	Np [atm^−05^]	T [°C]	t [h]	Np [atm^−05^]				
3A; 4A	580	5	3.20	600	11	0.50	NH_3_/NH_3_zd	-	-	-
13; 14	580	5	3.20	600	11	0.50	NH_3_/NH_3_zd	520	4	N_2_/Ar/P = 150 Pa

**Table 3:** The phase composition of nitride layer (white layer) and their thickness found for processes.

Grade Steel	Sample No.	Φ (mm)			
			g_mp_ (µm)	g_por_ (µm)	PC WL
AISI 1010	3A	3.0	50 ± 1	20 ± 1	Fe_4_N-γ’
AISI 1010	13	3.0	56 ± 1	25 ± 1	Fe_4_N-γ’; Fe-α
AISI 52100	4A	2.5	37 ± 1	11 ± 1	Fe_4_N-γ’
AISI 52100	14	2.5	38 ± 1	10 ± 1	Fe_4_N-γ’; Fe-α

g_mp_—nitride layer thickness; g_por_—thickness of the porous zone.

**Table 4:** The ratio, *D*, of the signal coming from the Dyson line into the signal coming from the Lorentz line.

Sample	Starting (SS)	Nitrided	Nitrided and Annealed
AISI 1010	0.23 *L*	0.74 *L*	0.38 *L*
AISI 52100	0.28 *L*	0.78 *L*	0.48 *L*

## Data Availability

The data could be found in the Westpomeranian University of Technology And Czestochowa University of Technology.
